# Ancestry Specific Polygenic Risk Score, Dietary Patterns, Physical Activity and Incident Type 2 Diabetes

**DOI:** 10.21203/rs.3.rs-7105280/v1

**Published:** 2025-08-21

**Authors:** Dale S. Hardy, Jane T. Garvin, Tennille S. Leak-Johnson, Tesfaye B. Mersha

**Affiliations:** BioNutriOmics, Inc; Walden University; Morehouse School of Medicine; Indiana University

**Keywords:** polygenic risk score, dietary patterns, DASH diet, Mediterranean diet, Southern diet, physical activity, interaction, type 2 diabetes, race, ancestry

## Abstract

**Background:**

It is unknown whether the impact of lifestyle could mitigate the genetically driven high-risk polygenic risk score (PRS) in different ancestries.

**Objectives:**

We determined associations and interactions between high-risk PRS, dietary patterns, physical activity, and metabolic burden and their impact on incident type 2 diabetes mellitus (T2DM) in European Americans and African Americans. A secondary aim determined ancestry-specific PRS-mapped genes associated molecular pathways.

**Methods:**

Our longitudinal study (1976–2015) utilized de-identified data for European American (n = 8,283) and African American (n = 1,205) from 7-National Heart, Lung, and Blood Institute Candidate Gene Association Resource studies from the Database of Genotypes and Phenotypes. We assessed results using biased-corrected odds ratios (OR) and 95% confidence intervals (CI).

**Results:**

African Americans had a higher magnitude of incident T2DM risk for the high-risk PRS alone (OR = 1.61; 95% CI:1.22–2.12) than European Americans (OR = 1.24; 95% CI:1.06–1.45) in highest tertile compared to lowest tertile. We observed protective risks from incident T2DM with the Dietary Approaches to Stop High Blood Pressure (DASH) and Mediterranean diets (p < 0.025). However, entangling effects from the high-quality DASH/Mediterranean diets with the low-quality Southern diet and the high-risk PRS further increased T2DM risks (p < 0.025). The PRS-diet-physical activity associations had 9% protective T2DM risk for Mediterranean diet in African Americans and at least 5% for European Americans (p < 0.025). Interactions revealed the second tertile DASH and highest tertile Mediterranean diets in high-risk PRS-high metabolic burden, attenuated incident T2DM risk. Gene enrichment molecular pathways common to ancestries included neurogenerative diseases, lipid metabolism, and glucose metabolism.

**Conclusions:**

Patients in the highest PRS tertiles with high metabolic burden could be targeted for early intervention to decrease T2DM risk. The DASH and Mediterranean diets with high physical activity should be recommended by clinicians for better prevention, detrimental molecular pathway reversal, and to decrease worsening of T2DM from high metabolic burden.

## INTRODUCTION

Diabetes is one of the fastest-growing noncommunicable diseases in the USA and worldwide that disproportionately affects people of color.^1^ Although the prevalence of diabetes is increasing, disparities remain for African Americans (17.4%) compared to European Americans (13.6%).^1^ According to Mitchell et al., there are disparities in cardiometabolic conditions and metabolic markers such as obesity, high blood pressure, HbA1c, etc., among African American adults compared to European Americans.^2^ The development of T2DM is driven by several key risk factors, including poor diet quality (Western or Southern diet),^3^ higher BMI values/obesity status, and abnormal values for clinical indices (blood glucose, blood pressure, and lipid levels, etc.), which play a major role in its diagnosis and progression.^2^ These risk factors have been known to interact with single nucleotide polymorphisms (SNPs) to increase cardiometabolic risk.^4,5^ Studies suggest that adherence to high-quality diets such as DASH and the Mediterranean diet, characterized by their emphasis on nutrient-dense foods, may be protective against type 2 diabetes and other cardiometabolic diseases.^6,7^ The low-quality Southern diet, in contrast to healthier eating patterns, elevates the risk of T2DM,^8^ as well as metabolic syndrome and coronary heart disease, both of which are linked to diabetes.^9^

The longitudinal influence of physical activity to attenuate the effects of a high-risk PRS on incident T2DM has not been well studied in African Americans. However, moderate to high physical activity, performed weekly as 150 to 300 minutes of moderate-intensity, or 75 to 150 minutes of vigorous-intensity aerobic physical activity, can reduce the risk of developing cardiometabolic disorders.^10^ A report demonstrated that increasing physical activity levels led to a progressively lower risk of T2DM, a relationship that persisted even when accounting for genetic risk.^11^ The benefits of physical activity, in terms of absolute risk reduction, were most pronounced in those with high genetic risk. In our cross-sectional study on atherosclerotic cardiovascular disease risk, we found that the lowering effects from high physical activity with the high-risk PRS, the high-quality DASH and Mediterranean diets and even the low-quality Southern diet decreased atherosclerotic cardiovascular disease risks by 9–15% in European Americans and by 13–18% for the DASH and Mediterranean diets in African Americans.^3^

The long-term impact of the genetic contribution of the PRS combined with high metabolic burden (clinical risk factors) on cardiometabolic disease risk in African Americans remains insufficiently studied. The purpose of this study was to examine the associations and interactions of the high-risk PRS with diet quality, physical activity, and metabolic burden and their impact on incident T2DM. A secondary aim was to determine the PRS-mapped genes associated with molecular variant characteristics and pathways between ancestries.

## METHODS

### Selection of Participants

This cohort study utilized data collected from 1976 to 2015 from seven parent studies from the National Heart, Lung, and Blood Institute (NHLBI) Candidate Gene Association Resource (CARe), which is part of the Database of Genotypes and Phenotypes (dbGaP) collection of studies.^12^ Clinical trial data were not used for this study. Only observational data were used from all seven NHLBI studies. We merged data from the Atherosclerosis Risk in Communities (ARIC) study,^13^ Coronary Artery Risk Development in Young Adults (CARDIA) study,^14^ Cardiovascular Heart Study (CHS),^15^ Framingham Heart Study (FHS) Offspring and GENX 3 studies,^16^ Multi-Ethnic Study of Atherosclerosis (MESA) study,^17^ and Women’s Health Initiative (WHI) study,^18^ to create a large dataset by ancestry in two racially diverse cohorts. All studies are large-scale, ongoing prospective cohort studies with atherosclerosis outcomes. Further design and sampling for all studies are described elsewhere.^13–18^

#### Consent to Participate Declaration

All participants signed an informed consent prior to participation in the parent study and agreed to take part voluntarily. The Morehouse School of Medicine Social & Behavioral Institutional Review Board (IRB) approved this study.

#### Human Ethics and Consent to Participate declarations

The parent studies were conducted with full ethical approval and informed consent, ensuring participants were adequately informed about the research objectives, methodologies, potential risks and benefits, and understood their right to voluntary participation. In addition, a statement regarding ethical approval from each NHLBI study’s IRB or ethics committee that included the IRB committee’s name and reference number was obtained.

#### Constructing the PRS and principal components for stratification

After SNPs were cleaned, we used the Michigan Imputation Server algorithm for 1000 Genomes Phase 3 (Version 5) to perform SNP imputation using dosage-mach algothrium.^19^ We then performed quality cleaning of each dataset, merging of imputed cleaned datasets, and extracted SNPs for the PRS using Plink, a whole genome association analysis toolset by ancestry.^20^ Only SNPs with an imputation quality (rsq) ≥ 0.3 passed quality control. We choose biallelic SNPs for our datasets for PRS calculation; however, most SNPs had minor allele frequencies ≥ 0.1. Tag SNPS in high linkage disequilibrium ≥ .8 with another SNP, were chosen based on higher binding capacity in RegulomeDB.^21^ The Hardy-Weinberg test for all SNPs were performed in unrelated samples of African Americans and European Americans separately in Plink^20^ using chi-square goodness-of-fit test. Ten genetic principal components were computed using Genome-wide Complex Trait Analysis (GCTA) guidelines^22^ to calculate a genetic-related matrix by ancestry and then specifying the principal components in RStudio.^23^

We selected SNPs associated with blood glucose with p < 0.05 by ancestry (European American or African American) independently. Ancestry was used as self-reported race based on evidence that these variables have been shown to be in high agreement.^24^ We flipped alleles for SNPs that had reduced T2DM risks, making their effects risk-raising. We used a lower RegulomeDB score which represented Transcription Factor (TF) Binding sites and matched TF motif plus chromatin accessibility peaks.^21^ RegulomeDB presents a scoring system with functional categories ranging from 1 to 6 that incorporates integrated annotations data on methylation, chromatin structure, protein motifs and binding. The lower RegulomeDB scores represents stronger evidence for a variant to be in a functional genomic region.^21^

We randomized combined datasets into train and test sets by ancestry in a 50:50 ratio but included all MESA observations in the trainset. We trained the PRS using a glucose phenotype. We computed the ancestry specific PRSs by summing the risk alleles corresponding to dietary patterns and T2DM, weighted by the effect size estimate of the risk variants identified from our genome-wide association datasets. We increased the predictive power of the PRS by the joint power of multiple SNPs in the PRS using the summary best linear unbiased prediction (SBLUP) method. We then generated summary statistics on the train dataset and replicated these statistics in the test dataset to create our high-risk PRS to use in our statistical models.

#### Study Variables

“We defined Type 2 Diabetes Mellitus (T2DM) as the outcome variable using American Diabetes Association criteria,^25^ identifying individuals with a diagnosis of T2DM, self-reported diabetes, fasting blood glucose levels at or above 126 mg/dL, non-fasting blood glucose levels at or above 200 mg/dL, or who were currently taking diabetes medications. Metabolic syndrome, a dichotomous variable representing metabolic burden, was created from the continuous values of body mass index, waist circumference, waist-to-hip ratio, systolic and diastolic blood pressure, high-density lipoprotein, low-density lipoprotein, triglycerides, total cholesterol, and fasting blood glucose. We used the first visit available as the baseline that included nutrition and physical activity data. T2DM and all covariates were measured in each study for: ARIC, CARDIA, CHS, FHS Offspring, FHS GENX 3, MESA, and WHI. Data visits is shown in **Supplemental Table 1**. Covariates considered for adjustment were age (dichotomized), sex at birth, physical activity (high/low), current smoking (yes/no), current drinking (yes/no), highest education level completed, a 5-categorical variable coded as elementary school, attended high school but did not graduate, graduated high school, attended college, and attended graduate school, and log-transformed total caloric intake, dichotomized. We carried forward or backward variables from previous or subsequent visits if there were missing observations, whichever time between was lesser.

### Data harmonization

To harmonize our data, we brought together data of varying formats (file formats, variable definitions, etc.) from the seven NHLBI Care datasets in order to generate a large cohesive dataset. All chosen variables were present in the NHLBI Care datasets. We transformed some variables to yes/no or high/low status to harmonize measures across datasets. For example, the physical activity variable had different formats across datasets. To harmonize the physical activity variable across datasets, we recoded this variable in high/low form by evaluating its functional form in each dataset. To enable our chosen variables to have consistent meaning across the 7-NHLBI Care datasets, we created variable definitions by using their existing variable definitions that agreed across all datasets.

#### Food frequency questionnaire assessment from multiple studies

Studies that used a semi-quantitative food frequency questionnaire (FFQ) to obtain information on dietary intake were ARIC,^26^ MESA,^27^ FHS studies,^28^ and WHI.^29^ Studies that used diet history recalls were CARDIA (FFQ for visit 2)^30^ and CHS.^31^ **Supplemental Table 1** shows more details of these studies. Generally, in the FFQ, participants reported their intake based on 9 levels of frequency, ranging from < 1 time per month to ≥ 6 times per day. In the diet history sessions, participants were asked questions about usual intake. At the examinations, interviewers showed participants standard serving sizes, typical servings using food models, and additional information to help estimate food intake such as brand names of prepared foods.

#### Dietary pattern construction from multiple studies

We created dietary patterns for DASH, Mediterranean, and Southern diets. These diets were constructed within each study dataset. **Supplemental Table 2** shows the nutrients and whole foods used in our dietary pattern scores along with their mean values by ancestry. We utilized the Nutrient Rich Factor (NRF) index, developed by Drewnowski et al.^32,33^ to assess nutrient density. This method calculates Hybrid Nutrient Density scores by referencing the Food and Nutrient Database for Dietary Studies. Specifically, for nutrients relevant to the DASH diet, nutrient density was determined by calculating the percentage of the Recommended Dietary Allowance (RDA) for each nutrient, weighted by overall caloric intake. Nutrient density is the ratio of nutrients to energy in food, typically expressed as the nutrient concentration per 100 kcal. It reflects intake of nutrients and food recommendations, such as protein, fiber, potassium, calcium, fruit, vegetables, wholegrain intake, sodium, sugar, etc. The NRF index comprehensively measures a food’s nutrient density, reflecting its contribution to overall diet quality.

Each nutrient and food was energy-adjusted using the residual method, standardized to a 2000 Kcal diet.^34^ All nutrients and foods used for the diet scores were present in each dataset. The DASH diet was constructed with nutrients such as vitamin D, calcium, magnesium, potassium, phosphorus, potassium to sodium ratio, thiamine, niacin, and fiber, etc. which provide integral benefits in lowering blood pressure.^6^ The Mediterranean diet consisted of whole foods that have been proven to lower the risk of T2DM. This includes fruits, vegetables, salads, nuts, wholegrains, beans and peas, chicken, and fish.^6,7^ Conversely, the Southern diet was composed of foods typically found in a Western diet, such as fried foods (French fries, fried chicken), sugar sweetened beverages (sodas), chips, red meats (hamburgers, pork), processed meats (deli meats, ham, hotdog, salami), organ meats (liver, kidneys), unlimited alcohol, sweets and desserts.^9^ The healthy DASH and Mediterranean diets represented diets with high-quality diet scores, and the harmful Southern diet, a low-quality diet score. After we assembled our foods for each diet, we added up the foods to compute each diet score.

### Statistical Analysis

Our longitudinal study was composed of 9,488 participants, in which 8,283 (87.30%) were European Americans and 1,205 (12.70%) were African Americans. To enhance the statistical power and representativeness of the sample, especially for African Americans, missing observations for cigarette smoking, drinking status, physical activity, and education level were imputed using the Stata mi imputation suite. Imputations were < 5% of the original participant sample. **Supplemental Fig. 1** shows how patients were selected into the study. Our initial unrelated sample without duplicates in identifiers was based on the PRS (n = 13,857). Our final models included 9,488 participants of which 8,283 (87.3%) were European Americans and 1,205 (12.7%) were African Americans. We did not drop missing observations across dietary patterns, because observations in dietary patterns were missing at random and would have decreased the sample size further within each dietary pattern.

We created a principal component adjusted PRS. In models that evaluated the effects of physical activity, and the PRS, and dietary patterns on incident T2DM risk, the physical activity variable was not included in the covariate summary score. To conserve power (especially for African Americans) and to decrease bias, we computed a covariate summary score by ancestry by regressing T2DM on the covariates. We used bias corrected Generalized Estimation Equation (xtgee) population-average panel-data models to derive bias-corrected odds ratios (OR) and 95% confidence (CI) intervals. In our xtgee analysis, we were interested in the expectation of the outcome T2DM, as a function of the PRS and/or specific dietary pattern (DASH, Mediterranean, Southern) and the covariate summary score to isolate the effect of the diet of interest.

In our multivariable models, we regressed T2DM on the PRS and each dietary pattern, adjusting for the covariate summary score. In interaction models, we included the PRS, each dietary pattern, along with the covariate summary score for adjustment. All models represented a 2-sided p < 0.05, and more stringent, Bonferroni adjustment for multiple testing (p < 0.025) were used as the threshold for statistical significance. All regression analyses were bootstrapped at least 10,000 times. Our multivariable statistical analyses were conducted using Stata MP, version 17.0 (StataCorp, College Station, TX).^35^

### Interaction between the PRS, dietary patterns, and physical activity on T2DM risk

In interaction analyses, we were interested in additive interaction in which the modifying effect of the dietary patterns elicit changes on different levels of the PRS, and how high physical activity affected this relationship. We computed the difference in probability for T2DM between the expected risks at lowest, second and highest tertiles of the PRS with tertiles in each dietary pattern in the presence of high and low physical activity adjusting for the covariate summary score.

### Pathway analysis of SNPs in PRS mapped to genes

We mapped the SNPs to their respective genes by ancestry, using SnpXplorer.^36^ SnpXplorer, a web-based application to explore human SNP-associations and annotate SNP-sets. SnpXplorer performs functional annotation and gene-set enrichment analysis on a provided list of SNPs. Consensus Pathway Analysis (CPA) was performed using the AIPA - AI Powered Pathway Analyzer, a web-based platform. This analysis involved Over-representation Analysis (ORA), as implemented in WebGestalt, to identify significantly impacted pathways.^37^ ORA translates gene lists into biological insights. We used ORA to compare the pathways that were common between the two gene sets from both ancestries with the expected overlap using a statistical T-test to determine significance using an adjusted p value.

## RESULTS

### The PRS was created for each genetic ancestry

We created PRSs by genetic ancestry separately for African Americans and European Americans. After merging the seven NHLBI Care imputed datasets, we obtained 5,957,358 markers each in European Americans and African Americans. After quality control procedures such as pruning and cleaning, and within Hardy-Weinberg equilibrium, 120,991 variants in European Americans and 265,042 variants in African Americans remained. Furthermore, after administering clumping and lasso shrinkage thresholding techniques, our datasets were reduced to 4598 variants in European Americans (n = 22,043) and 2067 variants in African Americans (n = 4036). After we randomized the participants into train and test datasets in a 50:50 ratio split and replicated the results of the train dataset into the test dataset, we identified a final list of and 224 SNPs from 382 genes in European Americans (n = 11,839) and 22 SNPs from 103 genes in African Americans (n = 2018). There was no overlap in genes between ancestries. The genotyping rates were 99.99% in European Americans and 100% in African Americans.

### Descriptive characteristics by ancestry at baseline

Our longitudinal study was composed of 9,488 participants, in which 8,283 (87.30%) were European Americans and 1,205 (12.70%) were African Americans. **Supplemental Table 3** shows the descriptive characteristics by ancestry. Unlike European Americans, a higher percentage of African Americans were obese, had proportion of obesity in Class I, II, and III obesity that were 1.5 to 2 times that of European Americans; were less physically active; had higher waist circumference; had higher systolic blood pressure, lower HDL cholesterol, higher fasting blood glucose levels, and a higher proportion were on blood pressure medications and diabetes medications. However, European Americans drank more alcohol and had higher triglyceride levels.

### PRS increased incident T2DM risk in each genetic ancestry

European Americans had an 18% elevated risk of incident T2DM (OR = 1.18; 95% CI:1.01–1.39) in tertile 2 vs. lowest tertile. However, the magnitude of risk in the highest tertile of 24% (OR = 1.24; 95% CI:1.06–1.45) compared to the lowest tertile did not meet that of African Americans (OR = 1.61; 95% CI:1.22–2.12) (Table 1). Most effect estimates mentioned passed the Bonferroni cut-off for false discovery rate at p < 0.025.

Type 2 diabetes status was regressed against the polygenic risk score, physical activity, and BMI adjusting for a covariate summary score composed of age, sex, current cigarette smoking status, current drinking status, education level, physical activity, time, and 10 principal components for population stratification.

### Physical activity decreased incident T2DM risk in each genetic ancestry

Table 1 shows the effects of physical activity on T2DM risk after adjusting for the covariate summary score. We observed a stable protective effect of 27% (OR = 0.73; 95% CI: 0.65–0.82) for European Americans. The physical activity estimate of 24% for African Americans was not as stable and did not pass did not pass the Bonferroni cut-off at p < 0.025.

### Dietary pattern effects decreased incident T2DM risk in each genetic ancestry

**Supplemental Fig. 2** shows the relationships over time for the different diets and incident T2DM (DASH, Mediterranean, and Southern diets). In general, the effect of the high-quality DASH and Mediterranean showed a protective effect against incident T2DM. There was a protective in European Americans in the 2nd tertile (OR = 0.68; 95% CI: 0.61–0.76) and 32% in the highest tertile in African Americans for the high-quality Mediterranean diet alone (OR = 0.68; 95% CI: 0.53–0.87). Interestingly, when the high-quality DASH or the Mediterranean diets were combined with the low-quality Southern diet, generally the risk for incident T2DM increased. This pattern was consistent among both ancestries. Alternately, when the Southern diet was combined with Mediterranean diet, there was an attenuation of increased incident T2DM risks. In European Americans, the highest tertile of adherence to a dietary pattern incorporating elements of both the DASH and Southern diets was associated with a 33% elevated risk compared to the lowest tertile (OR = 1.33; 95% CI: 1.20–1.48). However a much a higher incident T2DM risk of 2.21 times more likely was seen in African Americans (OR = 2.21; 95% CI: 1.74–2.81). African Americans had increased T2DM risks for the second tertile DASH combined with the highest tertile Southern diets (OR = 1.64; 95% CI: 1.21–2.23), and highest tertile DASH diet combined with second tertile Southern diet (OR = 1.67; 95% CI: 1.24–2.26). The low-quality Southern diet showed risk-raising effects for incident T2DM. We observed 40% increased risk in the highest tertile compared to the lowest tertile for European Americans (OR = 1.40; 95% CI: 1.28–1.54) and even more elevated T2DM risk of 98% increase for African Americans (OR = 1.98; 95% CI: 1.62–2.43).

### Associations between PRS and dietary patterns and incident T2DM risk by genetic ancestry

**Figure 1:**
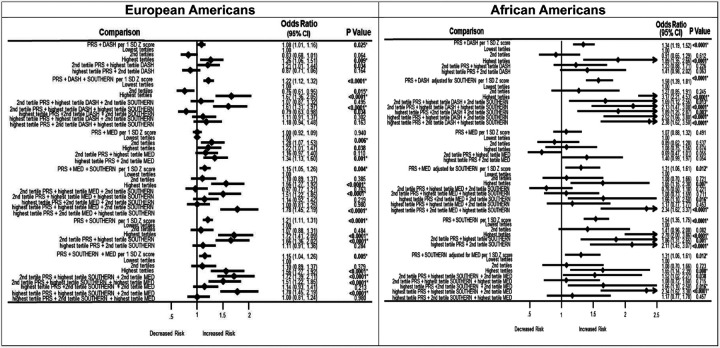
Association between PRS and dietary patterns, on incident type 2 diabetes. **Abbreviations:** PRS, polygenic risk score; DASH diet, Dietary Approaches to Stop High Blood Pressure diet; MED, Mediterranean diet. Bold indicates p values that were statistically significant at p < 0.05. *Bonferroni adjustment for multiple testing (p < 0.05/2= < 0.025). Type 2 diabetes status was regressed against each diet adjusting for a covariate summary score composed of age, sex, current cigarette smoking status, current drinking status, educational level, physical activity, and 10 genetic principal components for stratification. Dietary pattern scores were computed as a measure of food quality using values that were assigned to foods, scaled to an individual’s caloric intake. The foods were then adjusted using the residual energy adjustment method, then the selected foods were added up to form the dietary patterns which were converted to Z scores for analysis.

We examined the association between the PRS, dietary patterns and incident T2DM risk as shown in Fig. 1. Overall, the DASH and Mediterranean diets were not able to nullify the risk-raising PRS effects associated with increased incident T2DM risks due to the entangling effects of the low-quality high-risk Southern diet. Furthermore the combination with the low-quality Southern diet further increased these risks. We observed in the highest PRS-DASH tertile combination, there was an increased incident T2DM risk for European Americans (OR = 1.26; 95% CI: 1.06–1.51) and an even more elevated risk in African Americans (OR = 1.89; 95% CI: 1.35–2.66). However, when the PRS was combined with the high-quality DASH or Mediterranean diets plus the low-quality Southern diet, we observed a protective effect in the 2nd tertile in European Americans (OR = 0.76; 95% CI: 0.61–0.95), but the Mediterranean diet in the highest tertile was not able to overcome the effects of the PRS in the highest tertile (OR = 1.67; 95% CI: 1.36–2.05). Similarly, African Americans had increased effects in the highest PRS-Mediterranean tertiles combined with the Southern diet (OR = 1.17; 95% CI: 2.21–4.53). As expected, the PRS-Southern diet combination had significant risks in the highest tertiles for European Americans (OR = 1.72; 95% CI: 1.41–2.09). However, these risks were higher in African Americans (OR = 2.78; 95% CI: 2.00–3.86). Other combinations of the 2nd or highest tertiles of the PRS-DASH/Mediterranean/Southern diet combinations showed that as the tertiles of PRS or Southern diet increased, T2DM risks became more elevated. For example, in African Americans, the highest tertile PRS-highest tertile Mediterranean diet-2nd tertile Southern diet had a less elevated risk (OR = 1.66; 95% CI: 1.10–2.50) compared to the combination with the highest tertile of the Southern diet (OR = 2.34; 95% CI: 1.62–3.37). Figure 1

#### Associations between PRS, dietary patterns, and physical activity decreased incident T2DM risk in each genetic ancestry

**Figure 2:**
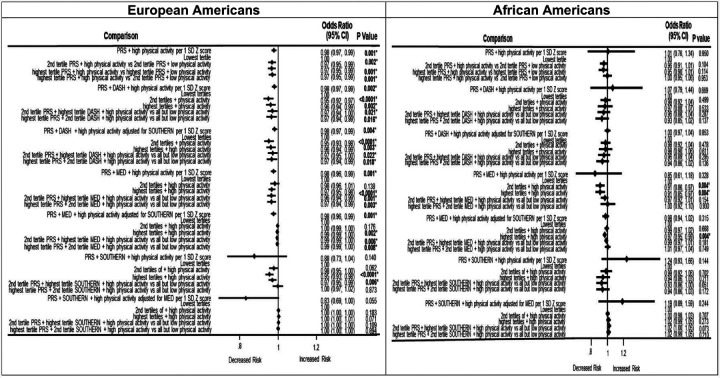
Association between PRS, dietary patterns, and physical activity on type 2 diabetes over time. **Abbreviations:** PRS, polygenic risk score; DASH diet, Dietary Approaches to Stop High Blood Pressure diet; MED, Mediterranean diet. Bold indicates p values that were statistically significant at p < 0.05. *Bonferroni adjustment for multiple testing (p < 0.05/2= < 0.025). Type 2 diabetes status was regressed against each diet adjusting for a covariate summary score composed of age, sex, current cigarette smoking status, current drinking status, educational level, physical activity, and 10 genetic principal components for stratification. Dietary pattern scores were computed as a measure of food quality using values that were assigned to foods, scaled to an individual’s caloric intake. The foods were then adjusted using the residual energy adjustment method, then the selected foods were added up to form the dietary patterns which were converted to Z scores for analysis

In Figure 2, we observed that physical activity consistently demonstrated protective effects within the PRS-diet-physical activity combinations. In European Americans, there was a 2% to 5% decreased risk in incident T2DM risk (p <0.0001). African Americans had less statistically significant results, but the estimates were in the direction of protective effects for incident T2DM. African Americans had 9% protective incident T2DM risk for PRS-Mediterranean diet-physical activity combination in the 2^nd^ (OR=0.91; 95% CI: 0.86–0.97) and highest (OR=0.91; 95% CI: 0.85–0.97) tertiles, and 3% decreased risk in the highest tertile of this same combination even when adjusted for the Southern diet (OR=0.97; 95% CI: 0.95–0.99).

#### Interaction between PRS, dietary patterns, and physical activity decreased incident T2DM risk in each genetic ancestry

**Figure 3:**
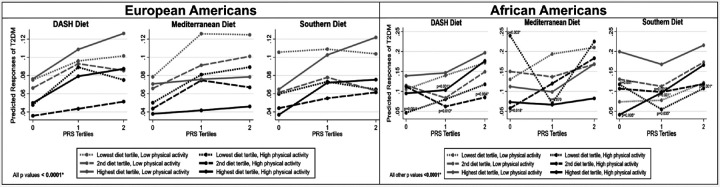
Predicted probabilities of type 2 diabetes over time for consuming different levels of dietary patterns (DASH, Mediterranean, and Southern) within tertiles of a polygenic risk score by low and high physical activity levels. Dietary patterns were converted to Z scores for analysis. *Bonferroni adjustment for multiple testing (p < 0.05/2= < 0.025).

Figure 3 shows the predicted probabilities of being diagnosed with T2DM when the DASH or Mediterranean diets adjusted for the Southern diet or the Southern diet adjusted for the Mediterranean diet were habitually consumed. When participants were habitually engaged in physical activity, the probability of being diagnosed with T2DM remained low. However, the Mediterranean diet in the highest tertile appeared to perform better than the DASH diet for both ancestries with low and high physical activity.

#### Interaction Between PRS, dietary patterns, and metabolic burden increased incident T2DM risk in each genetic ancestry

**Supplemental Table 4** shows the results of the combined obesity markers, clinical indices, and the combined obesity markers plus clinical indices adjusting for a covariate summary score. African Americans generally had higher incidence of T2DM risks compared to European Americans. The highest T2DM risks were seen in the combined effects of clinical indices. African Americans in the highest tertile of clinical indices had 5.57 times the risk compared to the lowest tertile (OR=5.57; 95% CI: 3.92–7.91); while European Americans had 4.19 times T2DM risk (OR=4.19; 95% CI: 3.55–4.93). The combined metabolic effects of obesity measures and clinical indices (metabolic burden) showed an almost doubling effect for African Americans (OR=9.94; 95% CI: 6.36–15.52) and European Americans (OR=7.87; 95% CI: 6.24–9.91) but incident T2DM risks for African Americans remained higher than European Americans.

**Figure 4:**
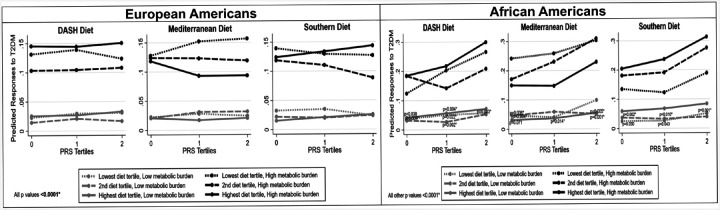
Predicted probabilities of type 2 diabetes over time for consuming different levels of diets (DASH, Mediterranean, and Southern) within tertiles of a polygenic risk score (PRS) by low and high metabolic burden. Metabolic burden were composed of body mass index, waist circumference, waist to hip ratio, systolic blood pressure, diastolic blood pressure, high density lipoprotein level, low density lipoprotein level, triglycerides, total cholesterol, and fasting blood glucose and then converted to Z scores for analysis. *Bonferroni adjustment for multiple testing (p < 0.05/2= < 0.025).

Figure 4 shows the predicted probabilities of being diagnosed with T2DM when the metabolic burden was high or low and patients consumed the high-quality DASH or Mediterranean diet adjusted for the low-quality Southern diet or the Southern diet adjusted for the high-quality Mediterranean diet. Individuals with low metabolic burden had decreased risk for incident T2DM regardless of PRS tertiles or diet quality. However, when the metabolic burden was high, the second tertile of DASH and highest tertile of Mediterranean diets were best in attenuating incident T2DM risks. Moreover, African Americans in the highest tertiles of metabolic burden experienced increased T2DM risks compared to those in the lower PRS tertiles, while European Americans showed a decreasing trend from lowest PRS tertile to highest PRS tertile.

#### Variant-Genetic differences and Pathways Common to Ancestries

**Supplemental Figure 3** shows the result of functional annotation of SNPs by ancestry. African Americans had a higher proportion of genes in chromosomes 7 and 17 than European Americans, but European Americans had higher proportion of genes in chromosomes 10, 11, and 12 than African Americans. European Americans had more gene overlap than African Americans. Applicable traits of interest in gene overlap common to ancestries was low density lipoprotein cholesterol. We further explored pathways common to ancestries in Consensus pathway Analysis. **Supplemental Figure 4** shows Overrepresentation Enrichment Analysis molecular pathways of the top 30 pathways that overlapped in gene enrichment between ancestries were for neurogenerative diseases, lipid metabolism and glucose metabolism.

## Discussion

Our study showed that African Americans had higher percentages of Class I, II, and III obesity. There were protective T2DM risks with the DASH and Mediterranean diets. However, entangling effects from the high-quality DASH/Mediterranean diets with the low-quality Southern diet and the high-risk PRS further increased T2DM risks. Furthermore, when PRS-diets were combined with physical activity, there were consistently small protective effects for incident T2DM. The DASH and Mediterranean diets were effective in alleviating high metabolic burden in the upper PRS tertiles for incident T2DM. Molecular pathways common to ancestries in gene enrichment were for neurogenerative diseases, lipid metabolism and glucose metabolism.

In this study, in general, African Americans consistently had 30% T2DM risks higher in magnitude of than European Americans. One encouraging occurrence we noticed is that when the low-quality Southern dietary pattern included foods from the high-quality Mediterranean diet, such as salads, we observed an attenuation of incident T2DM risk in the highest tertile. Conversely, the entangling effects with the high-quality DASH and Mediterranean diets and the low-quality Southern diet may increase T2DM risk, especially those patients in the highest tertile of the high-risk PRS. Physical activity had small consistent protective effects with the combination of the PRS-DASH or Mediterranean or Southern diets, when the PRS-diet combination were not able to nullify the risk-raising effects of the PRS.

Our study showed that obesity indices, high clinical indices, and high metabolic burden were associated with higher incident T2DM risks. In our published study, we observed that BMI was the best discriminator of incident T2DM among White males, Black males, and White females, while waist to hip ratio was the best discriminator of incident T2DM among Black females.^38^ In our cross-sectional study, we found that African Americans had almost doubled the rate of T2DM compared to European Americans, and this was mirrored in their obesity prevalence.^39^ In this study, African Americans’ intake of sugar-sweetened beverages over time was almost three times that of European Americans (**Supplemental Table 2**). Regular consumption of sugar-sweetened beverages (SSBs) significantly increases the risk of developing obesity, type 2 diabetes, and other conditions affecting the heart and metabolism.^40^ Because incident T2DM risks were consistently higher in African Americans, there is a need for new intervention studies with a different strategic focus in this population to decrease T2DM risks.

When the metabolic burden was high, the DASH and Mediterranean diets were efficacious in attenuating T2DM risks. However, while European Americans with high metabolic burden showed a decreasing trend from lowest to highest PRS tertile (Figure 4), African Americans showed an increasing metabolic burden trend but still had lowest incident T2DM risks in the second DASH and highest Mediterranean diet tertiles. Molecular pathways common to ancestries for neurogenerative diseases, lipid metabolism, and glucose metabolism are associated with the Southern dietary pattern. The Mediterranean diet is rich in antioxidants, fiber, and omega-3 polyunsaturated fatty acids, and the DASH diet has been reported to boost neurogenerative health, decrease coronary artery disease and T2DM incidence.^3,7–9^

Our study has several strengths, but few limitations. A major advantage of this study is that we were able to study incident T2DM risks over time that spanned almost 30 years with repeated measurements for diet, physical activity, and covariates. The large sample size enabled us to examine of incident T2DM representation from several different communities in the USA, which enhanced generalizability, especially for African Americans. Another strength was that we were able to impute variants from the large 1000 genomes reference database to augment our sample of variants available for the PRS.^19^ Our sample size decreased after dividing the genetic data into train and test sets. Recall bias and information bias were diminished because of the protocols established for collection of repeated measures of food frequency questionnaires and phenotypic information together. In addition, we used biased-corrected statistical models and did not see significant differences between results from models using odds ratios vs. risk ratios; we therefore subsequently used the simpler odds ratio models. Furthermore, we decreased bias for total caloric intake in food recall by dropping men and women who had outlier values that were above or below 1% percentile of total calories. Moreover, bias was diminished by converting the PRS, dietary pattern values, and metabolic burden values to Z-scores to standardize values across datasets.

An important limitation is the small sample size for the African American sample compared to the much larger sample of European Americans and, therefore, had less power to detect statistically significant associations. Most of the results that passed Bonferroni correction (p < 0.025) for African Americans were in the highest tertile vs. the lowest tertile; while European Americans frequently had more statistically significant estimates in the second and highest tertiles compared to the lowest tertiles. Nevertheless, we believe our results are an important contribution to science.

In conclusion, our study showed that the DASH and Mediterranean diets can modify the effects of a genetically high-risk PRS to attenuate risk for incident T2DM, but consuming large amounts of foods from the low-quality Southern diet can reposition a person from a protective effect to be at risk for incident T2DM. Because African Americans had higher prevalences of obesity, this suggests that our study may have broad application for those meeting the criteria for higher classes of obesity. For African Americans with high metabolic burden in particular should be monitored closely by clinicians for signs associated with risk for future T2DM and worsening of T2DM. The protective effects from physical activity over the course of this study were consistent, but small suggesting we need more types of studies on different types and duration of physical activity in different ancestries to decipher protective T2DM risks. Taken together, our findings show that high metabolic risk in the highest PRS tertile can be identified for preventive efforts. The high-quality DASH and the Mediterranean diets together with high physical activity should be recommended by clinicians to T2DM patients early for molecular reversal of harmful pathway effects, better prevention and to decrease complications and worsening of T2DM.

## Supplementary Material

This is a list of supplementary files associated with this preprint. Click to download.

- SUPPLEMENTALFIGURES.docx
- SUPPLEMENTALTABLES.docx

## Figures and Tables

**Table 1:** Association between a polygenic risk score, physical activity, body mass index, and type 2 diabetes over time.

Odds Ratio (95% Confidence Interval) P Value				
	European Americans	P Value	African Americans	P Value
	(n = 8,283)		(n = 1,205)	
**Polygenic risk score**				
PRS per 1 SD Z score	1.09 (1.02–1.17)	**0.016** *	1.27 (1.13–1.42)	**<0.0001** *
PRS lowest tertile	1.00		1.00	
PRS second tertile	1.18 (1.01–1.39)	**0.032**	1.04 (0.77–1.39)	0.800
PRS highest tertile	1.24 (1.06–1.45)	**0.005** *	1.61 (1.22–2.12)	**0.001** *
**Physical activity**				
Physical activity (high vs. low)	0.73 (0.65–0.82)	**<0.0001** *	**0.76 (0.57–1.00)**	**0.047**

Bold indicates p values that were statistically significant at p < 0.05.

* Bonferroni adjustment for multiple testing (p < 0.025/2 = < 0.025).

## Data Availability

The datasets used for the analyses described in this manuscript were obtained from dbGaP (https://www.ncbi.nlm.nih.gov/gap/) through dbGaP accessions. The data is available from dbGaP upon request. See **Supplemental Table 5** for the NHLBI CARe studies used for analysis.
